# Trends in body mass index among ever-married Bangladeshi women, 2004–14: evidence from nationally representative population-based surveys

**DOI:** 10.1017/jns.2021.1

**Published:** 2021-04-22

**Authors:** Md. Rafiqul Islam, Md. Sabbir Hossain, Md. Mostaured Ali Khan, Md. Shafiur Rahman

**Affiliations:** 1 Department of Population Science and Human Resource Development, University of Rajshahi, Rajshahi 6205, Bangladesh; 2 Department of Business Administration, Bangladesh Islami University, Dhaka, Bangladesh; 3 Department of Global Health Policy, School of International Health, The University of Tokyo, Tokyo, Japan; 4 Research Center for Child Mental Development, Hamamatsu University School of Medicine, Shizuoka, Japan

**Keywords:** Bangladesh Demographic and Health Surveys (BDHS), Body weight status, Bangladeshi women, Body mass index (BMI), Trends, Projection, Polynomial model

## Abstract

Both high and low body weight are associated with adverse health risk for both mother and children. Studies evaluating trends in the coverage of undernutrition and overnutrition among ever-married Bangladeshi women are limited. The objective of the present study is to assess the trends and develop future projections of body weight status among Bangladeshi women and to estimate the smoothed mean BMI by women's age for the national level and across urban and rural areas. Data from Bangladesh Demographic and Health Surveys conducted between 2004 and 2014 were used. The annual rate of change in the prevalence of underweight, overweight, and obesity, and smoothed age-specific mean BMI was estimated. During 2004–14, the prevalence of underweight reduced with an annual rate of 5⋅9 % at the national level, while the prevalence of overweight and obesity increased with an annual rate of 8⋅6 and 9⋅6 %, respectively. With the recent trends, the prevalence of underweight is expected to reduce from 11⋅9 % in 2020 to 6⋅5 % by 2025. In 2020, the prevalence of overweight and obesity were 30⋅0 and 6⋅9 %, respectively, which are projected to increase to 38⋅5 and 9⋅0 %, respectively, by 2025, if present trends continue. By 2030, the prevalence of overweight was predicted to be much higher in urban areas (44⋅7 %) compared with rural areas (36⋅5 %). Multifaceted nutrition programme should be introduced for rapid reduction of undernutrition and to halt the rise of the prevalence of overweight and obesity.

## Introduction

Both high and low body weight are the leading causes of death and disability in South Asian countries^(1)^. The slower reduction of undernutrition and the rapid increase of overweight and obesity have become a major challenge in the present century. Both underweight and overweight/obesity among women have consequences to their health as well as have intergenerational effects. For instances, low body mass index (BMI) or underweight increases the risk factor of hip fracture among women in their old age^(2)^. In addition, it is also associated with a higher risk of maternal mortality, infant mortality, premature birth, low birth weight, and other adverse birth and health outcomes^(3–5)^. Moreover, maternal pre-pregnancy underweight is associated with higher risk of malnutrition^(6)^ and delayed neurodevelopment among children^(6,7)^. Similarly, high BMI – overweight and obesity – is a vital risk factor for various chronic diseases such as coronary heart disease^(8)^, hypertension^(9)^, stroke, cancer, respiratory problems^(10,11)^ and diabetes mellitus^(12)^. A previous study mentioned that one unit decrease in BMI reduces the incidence of diabetes mellitus by 12⋅4–13⋅0 %^(13)^. A meta-analysis found that overweight or obese mother has increased risk of adverse birth and health outcomes including pre-eclampsia, gestational diabetes, gestational hypertension and delivery by a caesarean section^(14)^. Another meta-analysis documented that pre-pregnancy obesity is associated with elevated risk of neurodevelopment disorders and behavioural problems^(15)^.

Globally, in 2016, 9⋅4 % of adults were underweight, while 39⋅2 % were overweight and 15⋅1 % were obese^(16)^. However, the prevalence of underweight is still alarming in the South Asian region, where more than one in each five women (20⋅9 %) was found to be underweight in 2016. Previous studies reported that the prevalence of underweight is decreasing in most of the low- and middle-income countries (LMICs), while the prevalence of overweight and obesity is increasing rapidly^(17)^. Alike other LMICs, maternal and childhood malnutrition is considered to be a public health problem in Bangladesh and the government of Bangladesh has taken several initiatives to tackle the problem. Elimination of all forms of malnutrition among women and children is one of the key targets of the Sustainable Development Goals (SDGs)^(18)^. Thus, it is essential to evaluate the trends in body weight status among Bangladeshi women and to assess whether the country is in on track to achieve the short-term and long-term nutritional targets. Few previous studies investigated the trends of undernutrition and overnutrition among Bangladeshi women^(19,20)^; however, none of those studies developed short- or long-term projection of those indicators. Along with this, although, age, early age at first marriage, and early age at first delivery have been reported as potential factors for low or high BMI^(21–23)^, the age-specific mean BMI for reproductive-aged Bangladeshi women, the national level and across urban and rural areas, has yet to be investigated. However, the estimation of age-specific mean BMI among reproductive-aged women might help policy makers to develop targeted public health interventions through identifying the age group in which majority of women experiencing low or high BMI than normal BMI. Thus, this study aimed to assess the trends of body weight status among Bangladeshi women and develop short-term projections. We additionally estimated the smoothed mean BMI by women's age for the national level and across urban and rural areas. We also fitted several polynomial models to allow for better prediction of BMI values among reproductive-aged women.

## Data and methods

### Source of data

Data from Bangladesh Demographic and Health Surveys (BDHS) conducted in 2004, 2007, 2011 and 2014 were used. BDHS are nationally representative surveys conducted under the authority of the National Institute of Population Research and Training (NIPORT) of the Ministry of Health and Family Welfare (MOHFW), Government of Bangladesh, with the technical support from ICF International of Calverton, Maryland, USA, and funding from The U.S. Agency for International Development (USAID). Nationally representative, probability samples of men and women were selected for interview using a two-stage, stratified cluster sample of households that included strata for rural and urban areas and for the seven administrative divisions of Bangladesh. The detailed methodology of the BDHS 2004, 2007, 2011 and 2014 including the data collection method, validation and reliability assessment are explained in the BDHS reports^(24–27)^.

### Sample size

As the BDHS had information on ever-married women aged 15–49 years and their children from the selected households, the present study was limited to ever-married adult women in the reproductive age (15–49 years). Although individuals aged ≥18 years are considered as adults, approximately 51 % of Bangladeshi women get married before 18 years of age^(28)^. Evidence suggests that reproductive-aged underweight and overweight women from LMICs are associated with adverse health and birth consequences including maternal mortality^(3,4,14)^. Thus, we included ever-married women aged 15–49 years as participants in the present study. The sample sizes in BDHS 2004, 2007, 2011 and 2014 were 11 440, 10 996, 17 640 and 17 863, respectively. We excluded respondents due to missing information on BMI and respondents with extreme BMI values (BMI ≤12 and ≥50 kg/m^2)^^(29)^. Moreover, we excluded women who were pregnant at the time of the survey because body weight increases swiftly during the pregnancy and shows both nutritional statuses of the pregnant women and growth of the fetuses and as a result, it minimises the specificity of the indicator^(29,30)^. In addition, we excluded women who gave birth within 2 months from the date of interview. After excluding respondents with missing values and extreme BMI values, the final sample size considered in this study were 10 334, 9997, 16 021 and 17 675 for BDHS 2004, 2007, 2011 and 2014, respectively.

### Outcome measure

The main outcome variable for this study was the BMI, which is an indicator of body composition and calculated as the weight in kilograms divided by the square of the height in metres (kg/m^2)^. The age-specific mean BMI of Bangladeshi women was calculated for each survey year, both at the national level and across urban and rural areas separately, after adjusting for probability sampling design. Women were classified as having underweight for BMI <18⋅50 kg/m^2^, normal weight for the BMI value between 18⋅5 and 24⋅99 kg/m^2^, overweight for BMI between 25⋅00 and <30⋅00 kg/m^2^ and obesity for BMI ≥30⋅00 kg/m^2^^(31)^.

### Statistical analysis

#### Trends and projection assessment

The rate of change in the percentage of BMI categories – underweight, normal weight, overweight and obesity – during 2004–7, 2007–11 and 2011–14 were calculated using the compound annual growth rate formula. Later, an average annual rate of change for the period 2004–14 was estimated from those three-compound annual rates of change. The average annual rate of change during 2004–14 was then used to project the percentage of BMI categories up to the year 2025.

#### Data smoothing

After plotting the age-specific mean BMI of Bangladeshi women for years 2004, 2007, 2011 and 2014, we observed some sort of unexpected distortions in the data and thus, these BMI data were smoothened using the smoothing method ‘4253H, twice’ in the Package Minitab Release 12.1^(32)^.

#### Polynomial models

As the age-specific smoothed mean BMI for total, urban and rural areas seemed to be non-linear, we applied *n*th degree polynomial models of the following form to fit the smoothed data.

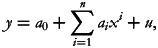

where *x* is the age (in year); *y* is the mean value of BMI; *a*_0_ is the constant; *a_i_* is the coefficient of *x^i^* (*i* = 1, 2, 3, …, *n*) and *u* is the disturbance term of the model with *u_i_* ~ NID(0, *σ*^2^)^(33)^. A suitable *n* is found for which the error sum of square is minimum. We used cross-validity prediction power (CVPP) to check how much these models are stable over population^(32)^. In addition, the *F*-test was used to verify the overall significance of the model. The statistical software STATISTICA was used to fit these mathematical models to mean BMI.

## Results

Table 1 presents the body weight status of Bangladeshi women in 2004, 2007, 2011 and 2014. About 18⋅4 % of total women, 12⋅8 % of urban women and 21⋅3 % of rural women in Bangladesh were underweight in 2014. However, about 33 % of women had underweight in 2004 at the national level, while 25⋅2 % of urban women and 37⋅6 % of rural women had underweight in 2004. A clear decreasing trend in the prevalence of underweight during 2004–14 was observed, the rate of reduction was 5⋅9, 6⋅5 and 5⋅6 % at the national level and across urban and rural areas, respectively. In addition, about 57⋅5 % of women had normal weight at the national level in 2014. The prevalence of normal weight has increased with an annual rate of increase of 0⋅2 and 0⋅6 % during 2004–14 at the national level and among rural areas, respectively, while the prevalence has decreased with an annual rate of 0⋅6 % among urban women during that period. On the other hand, about 8⋅8 % of women at the national level were overweight in 2004, which increased to 19⋅7 % by 2014 with an annual rate of increase of 8⋅6 %. The annual rate of increase of overweight during 2004–14 was even higher in rural areas (12⋅1 %) than urban areas (5⋅7 %). Similarly, only 1⋅8 % of Bangladeshi women were obese in 2004. However, the prevalence of obesity increased to 4⋅4 % by 2014 (with an annual rate of increase of 9⋅6 %). The prevalence of obesity was higher in urban areas (7⋅9 % in 2014) than that of rural areas (2⋅6 %).

**Table 1.** Nutritional status of reproductive-aged women at the national level and across urban and rural areas of Bangladesh, 2004–14

	Survey year				Average annual rate of change, 2004–14 (%)
	2004	2007	2011	2014	
National level					
Underweight	3442 (33⋅31 %)	2870 (28⋅71 %)	3778 (23⋅58 %)	3249 (18⋅38 %)	−5⋅9
Normal weight	5797 (56⋅1 %)	5749 (57⋅51 %)	9385 (58⋅58 %)	10 161 (57⋅49 %)	0⋅2
Overweight	911 (8⋅82 %)	1149 (11⋅49 %)	2345 (14⋅64 %)	3487 (19⋅73 %)	8⋅6
Obese	184 (1⋅78 %)	229 (2⋅29 %)	513 (3⋅2 %)	778 (4⋅4 %)	9⋅6
Total	10 334 (100 %)	9997 (100 %)	16 021 (100 %)	17 675 (100 %)	
Urban					
Underweight	897 (25⋅21 %)	783 (20⋅5 %)	869 (15⋅44 %)	777 (12⋅77 %)	−6⋅5
Normal weight	1954 (54⋅92 %)	2142 (56⋅07 %)	3160 (56⋅13 %)	3155 (51⋅85 %)	−0⋅6
Overweight	565 (15⋅88 %)	708 (18⋅53 %)	1259 (22⋅36 %)	1670 (27⋅44 %)	5⋅7
Obese	142 (3⋅99 %)	187 (4⋅9 %)	342 (6⋅07 %)	483 (7⋅94 %)	7⋅3
Total	3558 (100 %)	3820 (100 %)	5630 (100 %)	6085 (100 %)	
Rural					
Underweight	2545 (37⋅56 %)	2087 (33⋅79 %)	2909 (28 %)	2472 (21⋅33 %)	−5⋅6
Normal weight	3843 (56⋅71 %)	3607 (58⋅39 %)	6225 (59⋅91 %)	7006 (60⋅45 %)	0⋅6
Overweight	346 (5⋅11 %)	441 (7⋅14 %)	1086 (10⋅45 %)	1817 (15⋅68 %)	12⋅1
Obese	42 (0⋅62 %)	42 (0⋅68 %)	171 (1⋅65 %)	295 (2⋅55 %)	14⋅5
Total	6776 (100 %)	6177 (100 %)	10 391 (100 %)	11 590 (100 %)	

*Note:* Number within parenthesis presents percentage.

**Fig. 1.**
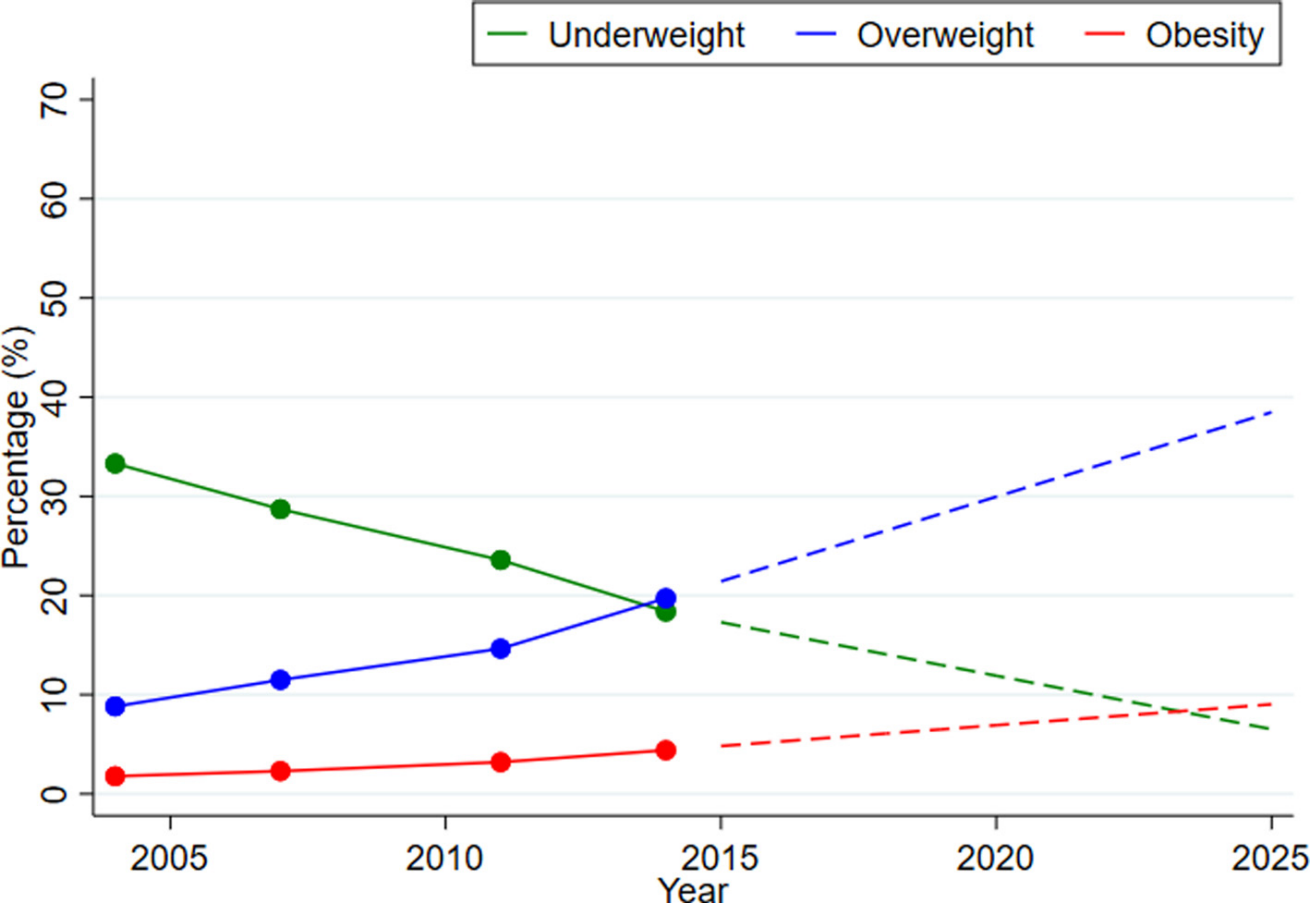
Trends and projections of body mass index among Bangladeshi women, 2004–25. *Note:* The coloured dots present the observed percentage and the dashed lines present projected trends in the future from 2014.

**Fig. 2.**
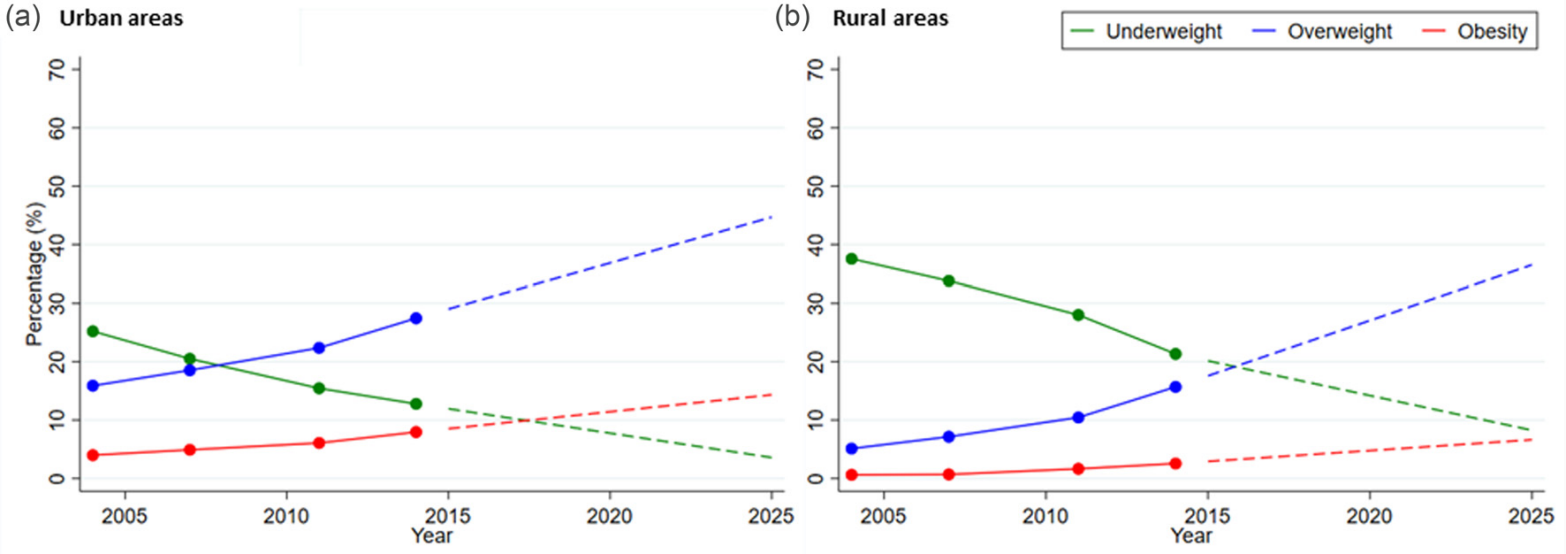
Trends and projections of body mass index among Bangladeshi women across (a) urban and (b) rural areas, 2004–25. *Note:* The coloured dots present the observed percentage and the dashed lines present projected trends in the future from 2014.

Using the observed rate of change during 2004–14, we also projected the prevalence of underweight, overweight and obesity from 2015 to 2025. We observed that the prevalence of underweight has reduced to 11⋅9 % at the national level by 2020 and it is predicted to be around 6⋅5 % by 2025 (Fig. 1 and Supplementary Table S6 of Supplementary material). On the contrary, the prevalence of overweight and obesity increased to 30 and 6 %, respectively, by 2020. By 2025, the prevalence of overweight and obesity is expected to be 38⋅5 and 9 %, respectively, if present trends continue (Fig. 1 and Supplementary Table S6 of Supplementary material). The prevalence of overweight and obesity is predicted to be much higher in urban areas (44⋅7 % for overweight and 14⋅3 % for obesity by 2025) compared to rural areas (36⋅5 % for overweight and 6⋅62 % for obesity) (Fig. 2 and Supplementary Table S6 of Supplementary material).

**Fig. 3.**
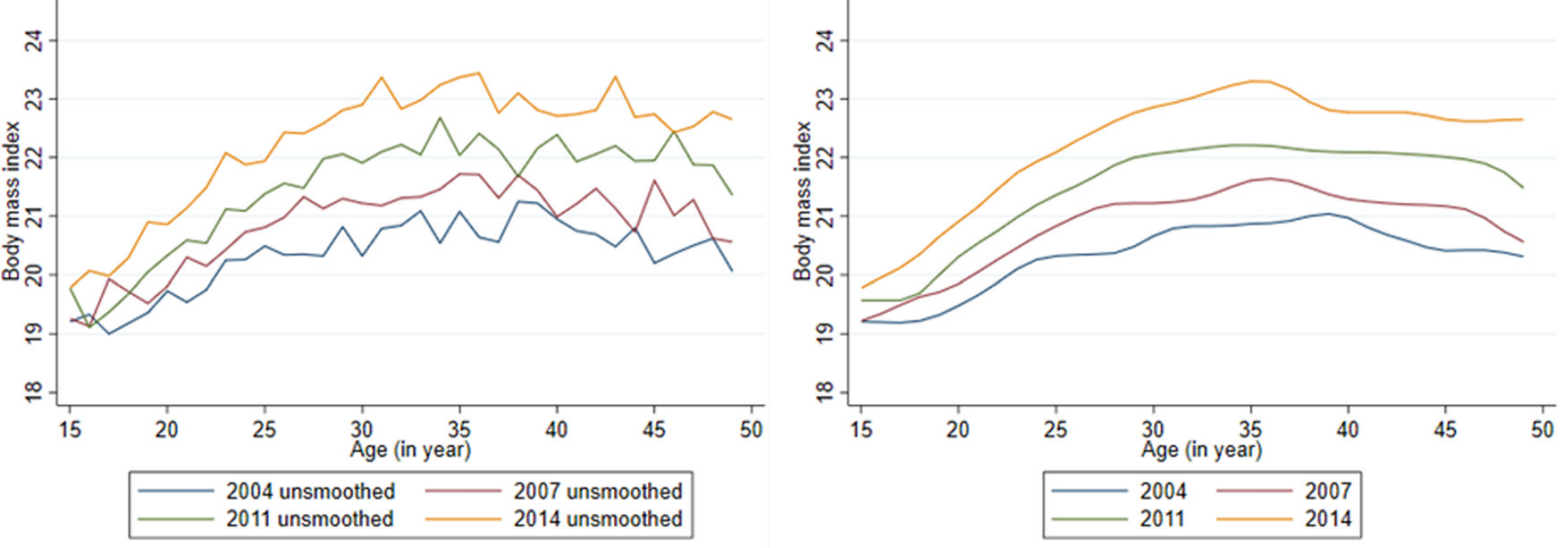
Age-specific unsmoothed and smoothed mean body mass index of ever-married Bangladeshi women at the national level. *Note:* The figure on the left shows the unsmoothed mean body mass index and right figure presents smoothed mean body mass index.

**Fig. 4.**
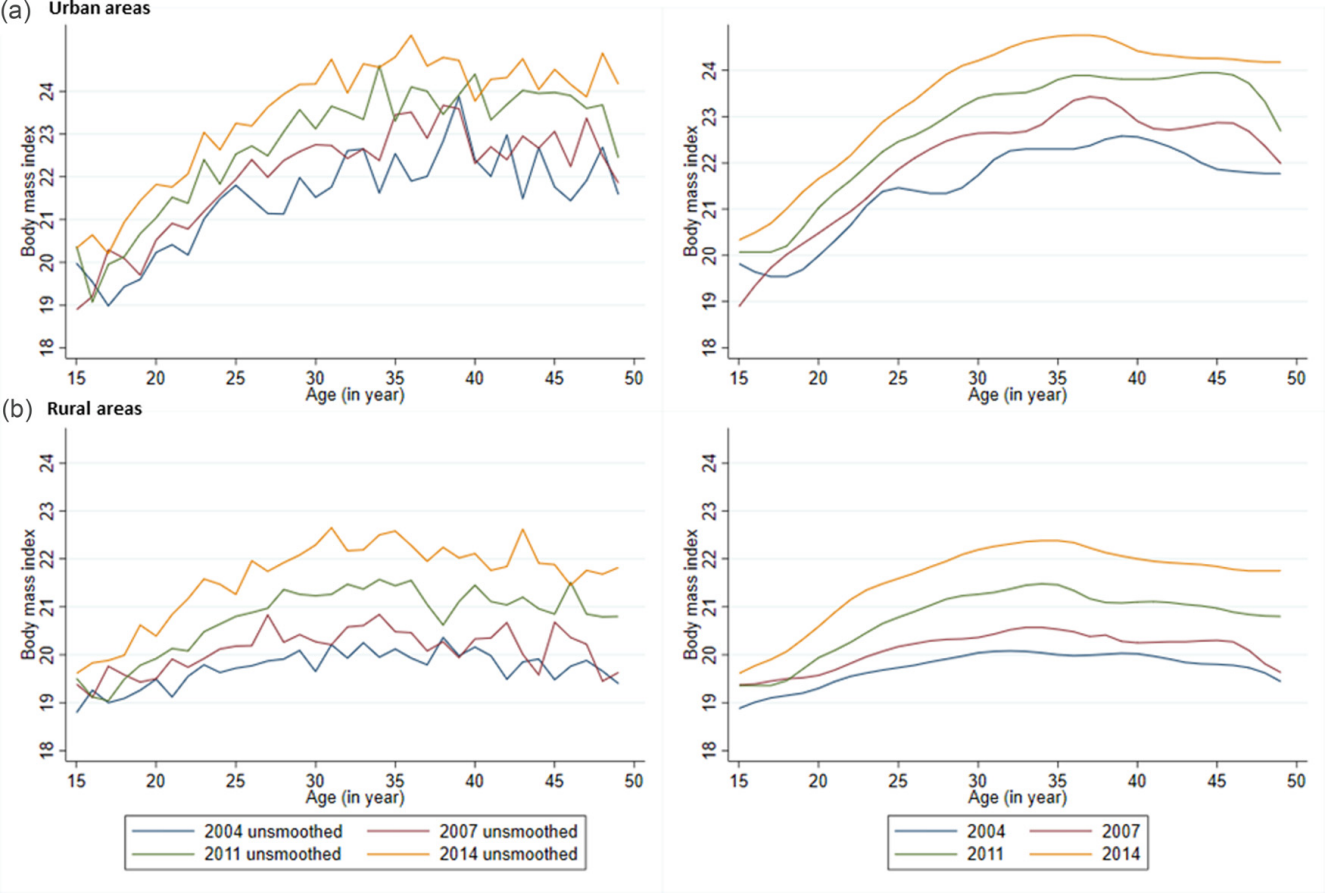
Age-specific unsmoothed and smoothed mean body mass index of ever-married Bangladeshi women across (a) urban and (b) rural areas. *Note:* The figures on the left show the unsmoothed mean body mass index and the figures on the right present smoothed mean body mass index.

The weighted mean BMI along with standard error at the national level, urban and rural areas in four consecutive surveys during the period 2004–14 are presented in Supplementary Tables S1–S4 of Supplementary material. The weighted mean BMI of women from urban areas was higher than that of women from rural areas at each age during 2004–14 except at age 17 years in 2004, at age 15 years in 2007 and at age 16 years in 2011. In addition, both unsmoothed and smoothed age-specific mean BMI for all four-survey years are presented in Figs. 3 and 4. For all four-survey years, the smoothed mean BMI was relatively low for women aged 20 years or younger (Table 2 and Fig. 3). Overall, a clear increase in age-specific mean BMI was observed during 2004–14 at the national level (Fig. 3) as well as in urban and rural areas (Fig. 4). In addition, the mean BMI increased with the increase of age, although a declining pattern was observed for age 45 years and over (Figs. 3 and 4). However, the highest BMI values were observed between age 30 and 40 years for the national level and urban and rural areas (Figs. 3 and 4). In addition, the smoothed mean BMI was higher in urban areas than rural areas for all ages and surveys (Fig. 4).

**Table 2.** Smoothed mean body mass index among reproductive-aged women at the national level, and across urban and rural areas of Bangladesh in 2004, 2007, 2011 and 2014

Age	2004			2007			2011			2014		
	Total	Urban	Rural	Total	Urban	Rural	Total	Urban	Rural	Total	Urban	Rural
15	19⋅21	19⋅82	18⋅88	19⋅22	18⋅89	19⋅37	19⋅57	20⋅07	19⋅36	19⋅78	20⋅33	19⋅61
16	19⋅20	19⋅64	19⋅01	19⋅34	19⋅34	19⋅39	19⋅57	20⋅07	19⋅36	19⋅95	20⋅49	19⋅77
17	19⋅19	19⋅54	19⋅10	19⋅49	19⋅73	19⋅45	19⋅57	20⋅07	19⋅36	20⋅12	20⋅69	19⋅90
18	19⋅22	19⋅54	19⋅15	19⋅63	20⋅02	19⋅50	19⋅69	20⋅20	19⋅46	20⋅35	21⋅01	20⋅07
19	19⋅32	19⋅69	19⋅20	19⋅71	20⋅25	19⋅52	20⋅00	20⋅59	19⋅70	20⋅65	21⋅37	20⋅32
20	19⋅48	19⋅99	19⋅30	19⋅85	20⋅48	19⋅57	20⋅31	21⋅03	19⋅94	20⋅91	21⋅66	20⋅59
21	19⋅66	20⋅31	19⋅44	20⋅05	20⋅72	19⋅68	20⋅54	21⋅35	20⋅09	21⋅16	21⋅88	20⋅88
22	19⋅87	20⋅65	19⋅55	20⋅26	20⋅95	19⋅82	20⋅75	21⋅62	20⋅26	21⋅46	22⋅16	21⋅15
23	20⋅10	21⋅07	19⋅62	20⋅46	21⋅23	19⋅96	20⋅98	21⋅93	20⋅46	21⋅74	22⋅53	21⋅35
24	20⋅26	21⋅38	19⋅68	20⋅66	21⋅57	20⋅07	21⋅19	22⋅24	20⋅65	21⋅93	22⋅88	21⋅48
25	20⋅32	21⋅46	19⋅73	20⋅83	21⋅86	20⋅17	21⋅36	22⋅46	20⋅78	22⋅09	23⋅13	21⋅59
26	20⋅34	21⋅40	19⋅78	20⋅99	22⋅10	20⋅23	21⋅51	22⋅59	20⋅90	22⋅28	23⋅35	21⋅70
27	20⋅35	21⋅34	19⋅85	21⋅13	22⋅30	20⋅29	21⋅68	22⋅77	21⋅03	22⋅45	23⋅63	21⋅83
28	20⋅37	21⋅34	19⋅91	21⋅21	22⋅47	20⋅32	21⋅87	22⋅99	21⋅16	22⋅62	23⋅91	21⋅95
29	20⋅48	21⋅46	19⋅97	21⋅22	22⋅58	20⋅33	22⋅00	23⋅22	21⋅23	22⋅76	24⋅10	22⋅09
30	20⋅66	21⋅73	20⋅04	21⋅22	22⋅64	20⋅36	22⋅06	23⋅40	21⋅26	22⋅86	24⋅21	22⋅19
31	20⋅79	22⋅07	20⋅07	21⋅24	22⋅65	20⋅43	22⋅10	23⋅48	21⋅30	22⋅93	24⋅34	22⋅26
32	20⋅83	22⋅26	20⋅08	21⋅28	22⋅64	20⋅52	22⋅14	23⋅50	21⋅37	23⋅02	24⋅50	22⋅31
33	20⋅83	22⋅30	20⋅07	21⋅37	22⋅68	20⋅57	22⋅18	23⋅52	21⋅45	23⋅13	24⋅62	22⋅36
34	20⋅84	22⋅30	20⋅04	21⋅50	22⋅83	20⋅57	22⋅21	23⋅63	21⋅48	23⋅23	24⋅69	22⋅38
35	20⋅87	22⋅30	20⋅00	21⋅61	23⋅11	20⋅53	22⋅21	23⋅80	21⋅46	23⋅30	24⋅74	22⋅38
36	20⋅88	22⋅30	19⋅98	21⋅64	23⋅35	20⋅48	22⋅20	23⋅89	21⋅34	23⋅29	24⋅76	22⋅34
37	20⋅92	22⋅37	19⋅99	21⋅60	23⋅43	20⋅38	22⋅16	23⋅89	21⋅17	23⋅16	24⋅76	22⋅23
38	21⋅00	22⋅51	20⋅01	21⋅49	23⋅39	20⋅41	22⋅12	23⋅84	21⋅09	22⋅95	24⋅72	22⋅13
39	21⋅04	22⋅58	20⋅03	21⋅37	23⋅19	20⋅28	22⋅10	23⋅81	21⋅08	22⋅81	24⋅58	22⋅06
40	20⋅97	22⋅56	20⋅02	21⋅29	22⋅90	20⋅25	22⋅09	23⋅81	21⋅10	22⋅77	24⋅42	22⋅00
41	20⋅81	22⋅47	19⋅97	21⋅25	22⋅74	20⋅26	22⋅09	23⋅81	21⋅11	22⋅77	24⋅35	21⋅95
42	20⋅68	22⋅35	19⋅91	21⋅22	22⋅71	20⋅27	22⋅08	23⋅84	21⋅09	22⋅77	24⋅32	21⋅92
43	20⋅58	22⋅20	19⋅84	21⋅20	22⋅75	20⋅27	22⋅06	23⋅90	21⋅05	22⋅77	24⋅28	21⋅90
44	20⋅47	22⋅00	19⋅81	21⋅19	22⋅81	20⋅29	22⋅04	23⋅95	21⋅02	22⋅72	24⋅26	21⋅88
45	20⋅41	21⋅86	19⋅80	21⋅17	22⋅87	20⋅30	22⋅01	23⋅95	20⋅97	22⋅65	24⋅26	21⋅84
46	20⋅42	21⋅82	19⋅78	21⋅12	22⋅86	20⋅27	21⋅97	23⋅90	20⋅89	22⋅62	24⋅24	21⋅78
47	20⋅42	21⋅79	19⋅73	20⋅97	22⋅68	20⋅09	21⋅90	23⋅72	20⋅84	22⋅62	24⋅20	21⋅75
48	20⋅38	21⋅77	19⋅62	20⋅74	22⋅36	19⋅81	21⋅75	23⋅32	20⋅81	22⋅64	24⋅18	21⋅75
49	20⋅31	21⋅77	19⋅44	20⋅56	21⋅98	19⋅63	21⋅48	22⋅68	20⋅80	22⋅65	24⋅18	21⋅75

The equation for 12 fitted polynomial models are presented in the Supplementary Appendix of Supplementary material and the model validation statistics are presented in Supplementary Table S7 of Supplementary material. All the fitted models from equation (1) to equation (12) are highly cross-validated and their shrinkages are very small, which indicate the better fit of the model. These polynomial equations will allow policy makers or other interested stakeholders to predict the BMI value for any age including fraction years of age.

## Discussion

The present study estimated trends of body weight status among Bangladeshi women aged 15–49 years at the national level and across urban and rural areas using nationally representative survey data and projected future prevalence of malnutrition indicators up to the year 2025 based on the current trends. To our knowledge, this is the first study to develop projection of such indicators, as well as, to provide age-specific smoothed mean BMI values using data from nationally representative population-based surveys in Bangladesh.

Around one in each eight women were found to be underweight in 2020, while approximately one-third of Bangladeshi women had overweight. The present study observed a slower reduction of underweight prevalence and a rapid increase in the prevalence of overweight and obesity, confirming a double burden of malnutrition. In particular, the prevalence of overweight and obesity in Bangladesh is increasing in an alarming rate. This finding is consistent with a prior study in Bangladesh^(34)^. The majority of the LMICs are now experiencing this nutritional transition, and Bangladesh is not an exception. A similar increase in the prevalence of overweight and obesity was also observed in neighbouring countries, such as India and Nepal^(35)^. In addition, our finding is also consistent with several previous studies that found increasing trends in the prevalence of overweight and obesity in LMICs^(36,37)^.

Our findings also highlighted that Bangladesh is likely to fail to control the uprising burden of overweight and obesity, although halting the rise of overweight and obesity by 2025 is one of the key nutrition-related targets adopted in the World Health Assembly in 2013^(38)^. This represents an urgent need to implement effective interventions in response to the rising burden of overnutrition. Otherwise, the country will face devastating public health consequences such as a rapid increase in the burden of non-communicable diseases. Lack of physical exercise, increased intake of unhealthy food and sweetened beverage in LMICs like Bangladesh are the key reasons for this increasing prevalence of overnutrition among women^(39,40)^. In line with our study, a recent study also reported that the probability of achieving the global target to halt the rise of obesity is almost zero^(17)^. That previous study found that none of the countries, out of included 193 countries, are projected to stop the increasing prevalence of obesity; while only 31 countries are on track to achieve the target for overweight^(17)^. However, the government of Bangladesh has taken multiple initiatives to reduce the spectrum of malnutrition, including the endorsement of the second ‘National Plan of Action for Nutrition (2016–2015)’^(41)^.

The present study found a higher prevalence of underweight among rural women, whereas the prevalence of overweight and obesity was always higher among urban women. This higher prevalence of overweight and obesity in urban areas might be due to the increased availability of unhealthy food and limited physical activity among urban residents^(42)^. The present study also observed that middle women aged (between 30 and 40 years) have relatively higher BMI than younger women. Thus, the community-level promotion of healthy lifestyles behaviours, especially among women aged in their 30's, could help to halt the rise of the overnutrition and obesity^(43,44)^.

### Strengths and limitations

The study has several strengths. The study was conducted based on the large sample size. In addition, this is one of the very few studies that has evaluated the recent trends and developed projections of nutritional indicators separately for urban and rural areas in Bangladesh. Moreover, this is the first study to provide smoothed age-specific mean BMI values for reproductive-aged women. The findings of this study would enable policy makers to identify the aged groups of women that are vulnerable to under- or overnutrition and thus help to formulate effective policies to address the burden of high or low BMI. However, the present study has certain limitations that need to be mentioned. First, the present study developed the projection of prevalence of underweight, overweight and obesity based on the past trends. However, it might not be robust as the future prevalence will depend on many other relevant factors, national policies, etc. Second, the present study was limited to ever-married women only, as nationally representative household surveys included only ever-married women. Thus, the findings cannot be generalised for all women of the reproductive age group. Lastly, polynomial models were not adjusted for other covariates.

## Conclusion

In conclusion, Bangladesh is presently facing dual burden of under- and overnutrition, where the burden of undernutrition is shifting towards overnutrition. The country is experiencing a slower reduction of undernutrition and a rapid increase in overnutrition among reproductive-aged women, which pose a significant challenge for Bangladesh to achieve the nutritional targets. Multifaceted nutrition programmes should be introduced for the rapid reduction of undernutrition and to halt the increase in the prevalence of overweight and obesity. Micronutrient interventions should be scaled up for the rapid reduction of undernutrition, and women in their 30's should be encouraged to adhere to healthy lifestyles behaviours to halt the rise of overweight and obesity.

## Supplementary material

For supplementary material accompanying this paper visit http://dx.doi.org/10.1017/jns.2021.1. click here to view supplementary material
