# Supplementary material for: Trends in body mass index among ever-married Bangladeshi women, 2004–14: evidence from nationally representative population-based surveys
Source: J Nutr Sci. 2021 Apr 22;10:e28. doi: 10.1017/jns.2021.1 (PMC8141677; doi:10.1017/jns.2021.1)
Supplement: Supplementary file 1 [file S204867902100001Xsup001.docx]

**Supplementary Table 1**: Sample size, weighted mean BMI and standard error of weighted mean BMI at total, urban and rural area of Bangladesh in 2004.

|  | Total | | | Urban | | | Rural | | |
| --- | --- | --- | --- | --- | --- | --- | --- | --- | --- |
| Age | Sample size | Weighted mean | S.E of mean | Sample size | Weighted mean | S.E of mean | Sample size | Weighted mean | S.E of mean |
| 15 | 162 | 19.09 | 0.20 | 57 | 20.12 | 0.57 | 105 | 18.76 | 0.18 |
| 16 | 229 | 19.38 | 0.17 | 62 | 19.99 | 0.72 | 167 | 19.26 | 0.14 |
| 17 | 285 | 19.05 | 0.11 | 80 | 19.02 | 0.30 | 205 | 19.05 | 0.12 |
| 18 | 274 | 19.17 | 0.14 | 73 | 19.56 | 0.36 | 201 | 19.08 | 0.15 |
| 19 | 330 | 19.31 | 0.13 | 98 | 19.64 | 0.37 | 232 | 19.23 | 0.14 |
| 20 | 401 | 19.67 | 0.13 | 130 | 20.17 | 0.33 | 271 | 19.54 | 0.14 |
| 21 | 369 | 19.37 | 0.14 | 121 | 20.43 | 0.39 | 248 | 19.07 | 0.13 |
| 22 | 418 | 19.59 | 0.13 | 140 | 20.13 | 0.31 | 278 | 19.44 | 0.14 |
| 23 | 351 | 20.17 | 0.17 | 135 | 20.88 | 0.37 | 216 | 19.93 | 0.19 |
| 24 | 385 | 20.14 | 0.15 | 134 | 21.65 | 0.39 | 251 | 19.64 | 0.15 |
| 25 | 390 | 20.26 | 0.15 | 146 | 21.79 | 0.41 | 244 | 19.76 | 0.14 |
| 26 | 376 | 20.23 | 0.17 | 127 | 21.67 | 0.40 | 249 | 19.83 | 0.17 |
| 27 | 355 | 20.31 | 0.18 | 135 | 21.45 | 0.42 | 220 | 19.90 | 0.19 |
| 28 | 354 | 20.17 | 0.16 | 120 | 21.10 | 0.39 | 234 | 19.91 | 0.17 |
| 29 | 349 | 20.69 | 0.18 | 136 | 22.05 | 0.38 | 213 | 20.21 | 0.19 |
| 30 | 322 | 20.08 | 0.17 | 117 | 21.33 | 0.42 | 205 | 19.65 | 0.17 |
| 31 | 310 | 20.62 | 0.21 | 119 | 21.92 | 0.50 | 191 | 20.16 | 0.21 |
| 32 | 341 | 20.51 | 0.20 | 116 | 22.71 | 0.49 | 225 | 19.92 | 0.21 |
| 33 | 370 | 20.97 | 0.21 | 131 | 23.14 | 0.55 | 239 | 20.34 | 0.20 |
| 34 | 321 | 20.40 | 0.18 | 113 | 21.52 | 0.42 | 208 | 20.07 | 0.20 |
| 35 | 313 | 20.91 | 0.21 | 125 | 22.54 | 0.48 | 188 | 20.30 | 0.22 |
| 36 | 279 | 20.40 | 0.20 | 101 | 21.64 | 0.49 | 178 | 20.00 | 0.21 |
| 37 | 260 | 20.33 | 0.22 | 92 | 21.95 | 0.55 | 168 | 19.83 | 0.22 |
| 38 | 298 | 20.92 | 0.20 | 107 | 22.64 | 0.50 | 191 | 20.35 | 0.20 |
| 39 | 273 | 20.80 | 0.24 | 87 | 23.94 | 0.64 | 186 | 20.04 | 0.23 |
| 40 | 294 | 20.66 | 0.23 | 104 | 22.35 | 0.55 | 190 | 20.13 | 0.23 |
| 41 | 229 | 20.57 | 0.25 | 87 | 22.03 | 0.62 | 142 | 20.07 | 0.25 |
| 42 | 220 | 20.42 | 0.29 | 76 | 22.74 | 0.69 | 144 | 19.69 | 0.30 |
| 43 | 223 | 20.27 | 0.24 | 85 | 21.57 | 0.62 | 138 | 19.85 | 0.25 |
| 44 | 199 | 20.58 | 0.25 | 64 | 22.65 | 0.67 | 135 | 20.05 | 0.25 |
| 45 | 233 | 20.03 | 0.26 | 74 | 21.76 | 0.66 | 159 | 19.59 | 0.27 |
| 46 | 240 | 20.19 | 0.24 | 86 | 21.28 | 0.63 | 154 | 19.87 | 0.25 |
| 47 | 191 | 20.46 | 0.30 | 59 | 21.81 | 0.87 | 132 | 20.13 | 0.30 |
| 48 | 202 | 20.27 | 0.26 | 64 | 22.65 | 0.73 | 138 | 19.67 | 0.25 |
| 49 | 188 | 19.71 | 0.26 | 57 | 21.40 | 0.72 | 131 | 19.27 | 0.26 |
| Total | 10334 | 20.16 | 0.03 | 3558 | 21.53 | 0.09 | 6776 | 19.76 | 0.03 |

**Supplementary Table 2:** Sample size, weighted mean BMI and standard error of weighted mean BMI at total, urban and rural area of Bangladesh in 2007.

|  | Total | | | Urban | | | Rural | | |
| --- | --- | --- | --- | --- | --- | --- | --- | --- | --- |
| Age | Sample size | Weighted mean | S.E of mean | Sample size | Weighted mean | S.E of mean | Sample size | Weighted mean | S.E of mean |
| 15 | 101 | 19.33 | 0.18 | 27 | 18.96 | 0.71 | 74 | 19.38 | 0.18 |
| 16 | 141 | 19.26 | 0.18 | 39 | 19.32 | 0.45 | 102 | 19.25 | 0.20 |
| 17 | 203 | 19.79 | 0.18 | 62 | 20.09 | 0.50 | 141 | 19.72 | 0.19 |
| 18 | 308 | 19.59 | 0.12 | 85 | 19.79 | 0.38 | 223 | 19.56 | 0.13 |
| 19 | 305 | 19.57 | 0.14 | 106 | 19.72 | 0.35 | 199 | 19.52 | 0.16 |
| 20 | 357 | 19.62 | 0.13 | 110 | 20.63 | 0.39 | 247 | 19.41 | 0.13 |
| 21 | 360 | 20.17 | 0.15 | 140 | 20.92 | 0.29 | 220 | 19.92 | 0.17 |
| 22 | 391 | 20.02 | 0.15 | 155 | 20.86 | 0.37 | 236 | 19.75 | 0.16 |
| 23 | 381 | 20.36 | 0.16 | 155 | 21.42 | 0.35 | 226 | 20.01 | 0.18 |
| 24 | 365 | 20.72 | 0.18 | 152 | 21.92 | 0.45 | 213 | 20.34 | 0.19 |
| 25 | 367 | 20.65 | 0.18 | 132 | 21.98 | 0.45 | 235 | 20.26 | 0.19 |
| 26 | 366 | 20.65 | 0.18 | 133 | 22.64 | 0.47 | 233 | 20.11 | 0.17 |
| 27 | 347 | 21.26 | 0.20 | 149 | 22.07 | 0.44 | 198 | 20.98 | 0.22 |
| 28 | 342 | 20.80 | 0.20 | 141 | 22.39 | 0.47 | 201 | 20.22 | 0.20 |
| 29 | 322 | 20.96 | 0.19 | 132 | 22.54 | 0.47 | 190 | 20.48 | 0.19 |
| 30 | 329 | 21.01 | 0.20 | 127 | 23.30 | 0.50 | 202 | 20.36 | 0.20 |
| 31 | 315 | 20.72 | 0.20 | 122 | 22.33 | 0.53 | 193 | 20.21 | 0.20 |
| 32 | 311 | 21.24 | 0.20 | 123 | 22.47 | 0.46 | 188 | 20.86 | 0.22 |
| 33 | 317 | 21.13 | 0.20 | 113 | 22.57 | 0.56 | 204 | 20.79 | 0.21 |
| 34 | 286 | 21.25 | 0.22 | 115 | 22.56 | 0.56 | 171 | 20.85 | 0.23 |
| 35 | 332 | 21.19 | 0.21 | 138 | 23.62 | 0.47 | 194 | 20.35 | 0.21 |
| 36 | 390 | 21.35 | 0.20 | 160 | 23.82 | 0.45 | 230 | 20.53 | 0.20 |
| 37 | 294 | 20.96 | 0.23 | 128 | 22.96 | 0.48 | 166 | 20.20 | 0.25 |
| 38 | 274 | 21.19 | 0.27 | 115 | 23.65 | 0.57 | 159 | 20.37 | 0.28 |
| 39 | 261 | 20.99 | 0.24 | 108 | 24.01 | 0.58 | 153 | 19.94 | 0.21 |
| 40 | 235 | 20.96 | 0.25 | 78 | 22.58 | 0.63 | 157 | 20.57 | 0.26 |
| 41 | 235 | 21.10 | 0.25 | 86 | 23.09 | 0.64 | 149 | 20.53 | 0.25 |
| 42 | 258 | 21.24 | 0.24 | 119 | 22.70 | 0.49 | 139 | 20.61 | 0.26 |
| 43 | 242 | 20.74 | 0.24 | 92 | 22.79 | 0.62 | 150 | 20.09 | 0.23 |
| 44 | 223 | 20.22 | 0.22 | 83 | 22.82 | 0.49 | 140 | 19.47 | 0.22 |
| 45 | 162 | 21.33 | 0.30 | 64 | 22.92 | 0.73 | 98 | 20.83 | 0.31 |
| 46 | 199 | 20.99 | 0.31 | 69 | 22.10 | 0.72 | 130 | 20.68 | 0.34 |
| 47 | 178 | 20.87 | 0.31 | 60 | 24.07 | 0.88 | 118 | 20.13 | 0.29 |
| 48 | 227 | 20.30 | 0.25 | 87 | 22.92 | 0.64 | 140 | 19.55 | 0.24 |
| 49 | 273 | 20.43 | 0.25 | 115 | 22.16 | 0.54 | 158 | 19.82 | 0.26 |
| Total | 9997 | 20.64 | 0.04 | 3820 | 22.25 | 0.09 | 6177 | 20.17 | 0.04 |

**Supplementary Table 3:** Sample size, weighted mean BMI and standard error of weighted mean BMI at total, urban and rural area of Bangladesh in 2011.

|  | Total | | | Urban | | | Rural | | |
| --- | --- | --- | --- | --- | --- | --- | --- | --- | --- |
| Age | Sample size | Weighted mean | S.E of mean | Sample size | Weighted mean | S.E of mean | Sample size | Weighted mean | S.E of mean |
| 15 | 119 | 19.75 | 0.20 | 38 | 20.55 | 0.63 | 81 | 19.54 | 0.19 |
| 16 | 212 | 19.11 | 0.14 | 68 | 19.08 | 0.30 | 144 | 19.12 | 0.16 |
| 17 | 272 | 19.27 | 0.14 | 99 | 19.98 | 0.31 | 173 | 19.02 | 0.14 |
| 18 | 426 | 19.75 | 0.13 | 130 | 20.49 | 0.29 | 296 | 19.52 | 0.14 |
| 19 | 465 | 20.04 | 0.13 | 146 | 20.81 | 0.32 | 319 | 19.80 | 0.14 |
| 20 | 562 | 20.26 | 0.14 | 208 | 21.22 | 0.26 | 354 | 19.89 | 0.16 |
| 21 | 497 | 20.40 | 0.13 | 163 | 21.28 | 0.29 | 334 | 20.08 | 0.15 |
| 22 | 632 | 20.38 | 0.13 | 226 | 21.43 | 0.27 | 406 | 19.98 | 0.13 |
| 23 | 608 | 20.90 | 0.14 | 202 | 22.38 | 0.33 | 406 | 20.42 | 0.15 |
| 24 | 593 | 21.07 | 0.14 | 223 | 21.94 | 0.27 | 370 | 20.71 | 0.16 |
| 25 | 639 | 21.20 | 0.14 | 216 | 22.73 | 0.33 | 423 | 20.73 | 0.15 |
| 26 | 642 | 21.54 | 0.15 | 240 | 23.01 | 0.28 | 402 | 20.95 | 0.16 |
| 27 | 602 | 21.45 | 0.15 | 202 | 22.72 | 0.31 | 400 | 21.00 | 0.17 |
| 28 | 590 | 21.81 | 0.16 | 216 | 23.04 | 0.32 | 374 | 21.36 | 0.17 |
| 29 | 553 | 21.90 | 0.15 | 190 | 23.77 | 0.32 | 363 | 21.26 | 0.16 |
| 30 | 566 | 21.81 | 0.16 | 200 | 22.96 | 0.30 | 366 | 21.38 | 0.18 |
| 31 | 555 | 21.88 | 0.17 | 194 | 23.74 | 0.33 | 361 | 21.23 | 0.18 |
| 32 | 490 | 22.21 | 0.18 | 179 | 23.98 | 0.33 | 311 | 21.54 | 0.21 |
| 33 | 458 | 21.88 | 0.18 | 159 | 23.41 | 0.40 | 299 | 21.37 | 0.20 |
| 34 | 433 | 22.47 | 0.21 | 159 | 24.80 | 0.39 | 274 | 21.63 | 0.23 |
| 35 | 556 | 21.96 | 0.16 | 179 | 23.32 | 0.33 | 377 | 21.53 | 0.19 |
| 36 | 422 | 22.13 | 0.18 | 141 | 24.25 | 0.42 | 281 | 21.46 | 0.19 |
| 37 | 410 | 21.93 | 0.19 | 152 | 24.21 | 0.36 | 258 | 21.01 | 0.20 |
| 38 | 403 | 21.35 | 0.18 | 150 | 23.49 | 0.37 | 253 | 20.52 | 0.19 |
| 39 | 416 | 22.12 | 0.21 | 154 | 24.55 | 0.46 | 262 | 21.20 | 0.21 |
| 40 | 618 | 22.37 | 0.18 | 198 | 25.03 | 0.38 | 420 | 21.48 | 0.19 |
| 41 | 413 | 21.81 | 0.18 | 153 | 23.46 | 0.38 | 260 | 21.19 | 0.20 |
| 42 | 406 | 21.70 | 0.20 | 157 | 23.52 | 0.39 | 249 | 20.90 | 0.20 |
| 43 | 354 | 22.03 | 0.22 | 126 | 24.60 | 0.43 | 228 | 21.20 | 0.23 |
| 44 | 317 | 21.65 | 0.22 | 104 | 24.38 | 0.51 | 213 | 20.81 | 0.22 |
| 45 | 425 | 21.64 | 0.20 | 150 | 24.20 | 0.40 | 275 | 20.81 | 0.22 |
| 46 | 331 | 22.33 | 0.27 | 130 | 24.42 | 0.65 | 201 | 21.50 | 0.25 |
| 47 | 335 | 21.58 | 0.23 | 126 | 23.56 | 0.48 | 209 | 20.82 | 0.25 |
| 48 | 382 | 21.63 | 0.23 | 143 | 23.87 | 0.51 | 239 | 20.78 | 0.23 |
| 49 | 319 | 21.28 | 0.23 | 109 | 22.42 | 0.52 | 210 | 20.88 | 0.25 |
| Total | 16021 | 21.39 | 0.03 | 5630 | 23.00 | 0.06 | 10391 | 20.82 | 0.03 |

**Supplementary Table 4:** Sample size, weighted mean BMI and standard error of weighted mean BMI at total, urban and rural area of Bangladesh in 2014.

|  | Total | | | Urban | | | Rural | | |
| --- | --- | --- | --- | --- | --- | --- | --- | --- | --- |
| Age | Sample size | Weighted mean | S.E of mean | Sample size | Weighted mean | S.E of mean | Sample size | Weighted mean | S.E of mean |
| 15 | 188 | 19.78 | 0.20 | 45 | 20.41 | 0.53 | 143 | 19.60 | 0.20 |
| 16 | 290 | 20.41 | 0.18 | 86 | 20.90 | 0.36 | 204 | 20.26 | 0.21 |
| 17 | 428 | 19.97 | 0.13 | 134 | 20.36 | 0.24 | 294 | 19.83 | 0.16 |
| 18 | 544 | 20.33 | 0.14 | 175 | 21.29 | 0.29 | 369 | 19.97 | 0.15 |
| 19 | 553 | 20.92 | 0.14 | 188 | 21.34 | 0.28 | 365 | 20.75 | 0.16 |
| 20 | 614 | 20.93 | 0.13 | 201 | 21.88 | 0.28 | 413 | 20.61 | 0.14 |
| 21 | 574 | 21.07 | 0.15 | 187 | 21.68 | 0.29 | 387 | 20.83 | 0.16 |
| 22 | 654 | 21.68 | 0.14 | 230 | 22.15 | 0.25 | 424 | 21.49 | 0.16 |
| 23 | 642 | 22.15 | 0.15 | 221 | 23.02 | 0.27 | 421 | 21.79 | 0.18 |
| 24 | 635 | 21.76 | 0.15 | 223 | 22.68 | 0.27 | 412 | 21.37 | 0.17 |
| 25 | 672 | 21.87 | 0.14 | 229 | 23.46 | 0.27 | 443 | 21.26 | 0.16 |
| 26 | 693 | 22.24 | 0.15 | 263 | 23.25 | 0.25 | 430 | 21.78 | 0.18 |
| 27 | 638 | 22.36 | 0.15 | 227 | 23.96 | 0.29 | 411 | 21.76 | 0.17 |
| 28 | 655 | 22.48 | 0.15 | 215 | 23.89 | 0.26 | 440 | 22.00 | 0.18 |
| 29 | 653 | 22.72 | 0.16 | 228 | 24.44 | 0.26 | 425 | 22.03 | 0.18 |
| 30 | 654 | 22.60 | 0.16 | 213 | 24.22 | 0.29 | 441 | 22.08 | 0.19 |
| 31 | 587 | 23.44 | 0.18 | 203 | 24.86 | 0.31 | 384 | 22.85 | 0.21 |
| 32 | 624 | 22.78 | 0.17 | 230 | 24.08 | 0.31 | 394 | 22.21 | 0.19 |
| 33 | 535 | 23.14 | 0.18 | 173 | 24.96 | 0.32 | 362 | 22.37 | 0.20 |
| 34 | 590 | 23.57 | 0.17 | 213 | 25.12 | 0.32 | 377 | 22.89 | 0.20 |
| 35 | 521 | 23.10 | 0.18 | 185 | 24.45 | 0.34 | 336 | 22.54 | 0.20 |
| 36 | 471 | 23.74 | 0.20 | 181 | 26.13 | 0.37 | 290 | 22.58 | 0.19 |
| 37 | 467 | 22.73 | 0.19 | 143 | 24.58 | 0.37 | 324 | 22.07 | 0.21 |
| 38 | 425 | 23.18 | 0.21 | 143 | 24.93 | 0.32 | 282 | 22.54 | 0.25 |
| 39 | 434 | 22.73 | 0.21 | 128 | 24.79 | 0.41 | 306 | 22.06 | 0.23 |
| 40 | 438 | 22.58 | 0.18 | 157 | 23.36 | 0.34 | 281 | 22.28 | 0.22 |
| 41 | 421 | 22.61 | 0.21 | 164 | 24.11 | 0.34 | 257 | 21.96 | 0.26 |
| 42 | 404 | 22.31 | 0.22 | 159 | 23.94 | 0.38 | 245 | 21.50 | 0.26 |
| 43 | 474 | 23.40 | 0.20 | 168 | 24.97 | 0.42 | 306 | 22.84 | 0.23 |
| 44 | 412 | 22.78 | 0.23 | 150 | 24.91 | 0.39 | 262 | 21.85 | 0.26 |
| 45 | 404 | 22.60 | 0.21 | 132 | 24.41 | 0.40 | 272 | 21.95 | 0.25 |
| 46 | 373 | 22.18 | 0.23 | 135 | 24.32 | 0.42 | 238 | 21.35 | 0.25 |
| 47 | 318 | 22.37 | 0.25 | 115 | 23.91 | 0.44 | 203 | 21.63 | 0.28 |
| 48 | 351 | 22.79 | 0.23 | 120 | 25.51 | 0.39 | 231 | 21.69 | 0.25 |
| 49 | 339 | 22.66 | 0.25 | 121 | 24.05 | 0.48 | 218 | 22.09 | 0.27 |
| Total | 17675 | 22.26 | 0.03 | 6085 | 23.66 | 0.06 | 11590 | 21.71 | 0.03 |

**Supplementary Table 5**: Unsmoothed mean BMI at total, urban and rural area of Bangladesh in 2004, 2007, 2011 and 2014

| Age | 2004 | | | 2007 | | | 2011 | | | 2014 | | |
| --- | --- | --- | --- | --- | --- | --- | --- | --- | --- | --- | --- | --- |
|  | Total | Urban | Rural | Total | Urban | Rural | Total | Urban | Rural | Total | Urban | Rural |
| 15 | 19.21 | 19.98 | 18.79 | 19.26 | 18.89 | 19.39 | 19.78 | 20.38 | 19.51 | 19.78 | 20.33 | 19.61 |
| 16 | 19.33 | 19.54 | 19.26 | 19.13 | 19.2 | 19.11 | 19.11 | 19.07 | 19.12 | 20.07 | 20.64 | 19.83 |
| 17 | 19 | 18.98 | 19 | 19.93 | 20.29 | 19.76 | 19.37 | 19.95 | 19.04 | 19.98 | 20.20 | 19.88 |
| 18 | 19.18 | 19.43 | 19.09 | 19.72 | 20.09 | 19.58 | 19.68 | 20.13 | 19.49 | 20.29 | 20.94 | 19.99 |
| 19 | 19.36 | 19.6 | 19.26 | 19.52 | 19.7 | 19.43 | 20.05 | 20.67 | 19.78 | 20.90 | 21.44 | 20.62 |
| 20 | 19.73 | 20.23 | 19.49 | 19.81 | 20.52 | 19.5 | 20.33 | 21.04 | 19.92 | 20.86 | 21.82 | 20.39 |
| 21 | 19.54 | 20.41 | 19.12 | 20.3 | 20.91 | 19.91 | 20.59 | 21.52 | 20.13 | 21.14 | 21.76 | 20.84 |
| 22 | 19.75 | 20.17 | 19.55 | 20.15 | 20.78 | 19.74 | 20.54 | 21.38 | 20.08 | 21.49 | 22.07 | 21.17 |
| 23 | 20.25 | 21.01 | 19.79 | 20.43 | 21.19 | 19.92 | 21.12 | 22.4 | 20.48 | 22.08 | 23.04 | 21.58 |
| 24 | 20.26 | 21.48 | 19.63 | 20.73 | 21.57 | 20.12 | 21.09 | 21.83 | 20.64 | 21.88 | 22.63 | 21.47 |
| 25 | 20.49 | 21.8 | 19.72 | 20.81 | 21.94 | 20.18 | 21.38 | 22.53 | 20.8 | 21.94 | 23.25 | 21.26 |
| 26 | 20.34 | 21.47 | 19.77 | 20.98 | 22.4 | 20.19 | 21.56 | 22.72 | 20.88 | 22.43 | 23.19 | 21.96 |
| 27 | 20.35 | 21.14 | 19.87 | 21.33 | 21.99 | 20.83 | 21.48 | 22.49 | 20.97 | 22.41 | 23.63 | 21.74 |
| 28 | 20.32 | 21.13 | 19.91 | 21.13 | 22.38 | 20.26 | 21.98 | 23.05 | 21.36 | 22.58 | 23.93 | 21.92 |
| 29 | 20.82 | 21.98 | 20.09 | 21.3 | 22.59 | 20.42 | 22.06 | 23.57 | 21.26 | 22.81 | 24.16 | 22.08 |
| 30 | 20.32 | 21.52 | 19.65 | 21.22 | 22.75 | 20.27 | 21.91 | 23.12 | 21.23 | 22.90 | 24.17 | 22.29 |
| 31 | 20.79 | 21.76 | 20.21 | 21.18 | 22.73 | 20.21 | 22.1 | 23.65 | 21.26 | 23.37 | 24.75 | 22.65 |
| 32 | 20.84 | 22.61 | 19.93 | 21.31 | 22.43 | 20.58 | 22.22 | 23.51 | 21.47 | 22.83 | 23.96 | 22.17 |
| 33 | 21.09 | 22.65 | 20.25 | 21.33 | 22.65 | 20.61 | 22.05 | 23.34 | 21.37 | 22.98 | 24.64 | 22.19 |
| 34 | 20.54 | 21.62 | 19.95 | 21.46 | 22.38 | 20.84 | 22.68 | 24.6 | 21.57 | 23.24 | 24.56 | 22.50 |
| 35 | 21.08 | 22.54 | 20.12 | 21.72 | 23.45 | 20.48 | 22.04 | 23.3 | 21.44 | 23.37 | 24.80 | 22.58 |
| 36 | 20.64 | 21.9 | 19.93 | 21.71 | 23.51 | 20.46 | 22.41 | 24.1 | 21.55 | 23.44 | 25.31 | 22.28 |
| 37 | 20.56 | 22.01 | 19.79 | 21.31 | 22.9 | 20.08 | 22.14 | 24 | 21.05 | 22.76 | 24.59 | 21.95 |
| 38 | 21.25 | 22.83 | 20.36 | 21.7 | 23.67 | 20.27 | 21.68 | 23.46 | 20.62 | 23.10 | 24.79 | 22.24 |
| 39 | 21.22 | 23.87 | 19.98 | 21.44 | 23.59 | 19.94 | 22.16 | 23.92 | 21.11 | 22.81 | 24.72 | 22.02 |
| 40 | 20.95 | 22.4 | 20.16 | 20.99 | 22.31 | 20.33 | 22.39 | 24.4 | 21.45 | 22.71 | 23.77 | 22.11 |
| 41 | 20.75 | 22.01 | 19.98 | 21.21 | 22.7 | 20.35 | 21.93 | 23.33 | 21.11 | 22.74 | 24.28 | 21.76 |
| 42 | 20.69 | 22.98 | 19.49 | 21.47 | 22.4 | 20.67 | 22.06 | 23.69 | 21.04 | 22.81 | 24.32 | 21.84 |
| 43 | 20.48 | 21.49 | 19.85 | 21.13 | 22.95 | 20.01 | 22.2 | 24.02 | 21.2 | 23.38 | 24.76 | 22.62 |
| 44 | 20.8 | 22.68 | 19.91 | 20.73 | 22.67 | 19.58 | 21.94 | 23.95 | 20.96 | 22.69 | 24.04 | 21.91 |
| 45 | 20.2 | 21.76 | 19.48 | 21.61 | 23.06 | 20.68 | 21.95 | 23.97 | 20.85 | 22.74 | 24.51 | 21.88 |
| 46 | 20.36 | 21.44 | 19.76 | 21.01 | 22.24 | 20.36 | 22.45 | 23.9 | 21.51 | 22.43 | 24.16 | 21.45 |
| 47 | 20.5 | 21.91 | 19.88 | 21.28 | 23.37 | 20.22 | 21.88 | 23.6 | 20.85 | 22.53 | 23.87 | 21.76 |
| 48 | 20.62 | 22.69 | 19.66 | 20.62 | 22.49 | 19.45 | 21.87 | 23.68 | 20.79 | 22.78 | 24.89 | 21.68 |
| 49 | 20.06 | 21.58 | 19.4 | 20.56 | 21.85 | 19.63 | 21.36 | 22.44 | 20.8 | 22.65 | 24.16 | 21.82 |

**Supplementary Table 6**: Projected prevalence of underweight, overweight, and obesity among Bangladeshi women

| **BMI categories** | **Prevalence by year** | | |
| --- | --- | --- | --- |
|  | **2015** | **2020** | **2025** |
| **National level** |  |  |  |
| Underweight | 17.30 | 11.91 | 6.52 |
| Normal weight | 57.62 | 58.26 | 58.90 |
| Overweight | 21.43 | 29.96 | 38.48 |
| Obese | 4.82 | 6.92 | 9.03 |
| **Urban** |  |  |  |
| Underweight | 11.93 | 7.76 | 3.58 |
| Normal weight | 51.52 | 49.89 | 48.26 |
| Overweight | 29.01 | 36.85 | 44.70 |
| Obese | 8.52 | 11.43 | 14.33 |
| **Rural** |  |  |  |
| Underweight | 20.14 | 14.20 | 8.25 |
| Normal weight | 60.84 | 62.77 | 64.71 |
| Overweight | 17.58 | 27.05 | 36.53 |
| Obese | 2.92 | 4.77 | 6.62 |

**Supplementary Appendix:**

Equations of fitted polynomial models are presented below.

(1) Polynomial model for national level, 2004:

Y = 15.20155 +0.30635 X - 0.00415 X^2^

t-stat (65.2348) (19.7386) (-17.2972)

P-value (0.0000) (0.0000) (0.0000)

(2) Polynomial model for urban area, 2004:

Y = 13.72321 – 0.44559 X - 0.00577 X^2^

t-stat (31.3692) (15.2930) (-12.8013)

P-value (0.0000) (0.0000) (0.0000)

(3) Polynomial model for rural area, 2004:

Y = 16.41237+0.20618 X - 0.00292 X^2^

t-stat (208.858) (39.3948) (-36.0151)

P-value (0.0000) (0.0000) (0.0000)

(4) Polynomial model for national level, 2007:

Y = 14.39116+0.38877 X –0.00533 X^2^

t-stat (77.2071) (31.3160) (-27.7463)

P-value (0.0000) (0.0000) (0.0000)

(5) Polynomial model for urban area, 2007:

Y = 11.69326+0.60146 X - 0.00791 X^2^

t-stat (38.3925) (29.6505) (-25.2126)

P-value (0.0000) (0.0000) (0.0000)

(6) Polynomial model for rural area, 2007:

Y = 16.51270+0.22542 X - 0.00322 X^2^

t-stat (88.5245) (18.1450) (-16.7382)

P-value (0.0000) (0.0000) (0.0000)

(7) Polynomial model for national level, 2011:

Y = 13.90402+0.442615 X –0.005857 X^2^

t-stat (58.3902) (27.9086) (-23.8683)

P-value (0.0000) (0.0109) (0.0008)

(8) Polynomial model for urban area, 2011:

Y = 12.51709 + 0.57802 X- 0.00732 X^2^

t-stat (35.6306) (24.7047) (-20.2331)

P-value (0.0000) (0.0000) (0.0000)

(9) Polynomial model for rural area, 2011:

Y = 14.83145+0.35860 X –0.00493 X^2^

t-stat (57.4728) (20.8641) (-18.5405)

P-value (0.0000) (0.0000) (0.0000)

(10) Polynomial model for national level, 2014:

Y = 13.46185+0.51480 X –0.00685 X^2^

t-stat (43.0654) (24.7274) (-21.2559)

P-value (0.0000) (0.0000) (0.0000)

(11) Polynomial model for urban area, 2014:

Y = 12.21092 + 0.64580 X- 0.00835 X^2^

t-stat (37.1511) (29.5010) (-24.6443)

P-value (0.0000) (0.0000) (0.0000)

(12) Polynomial model for rural area, 2014:

Y = 14.33886+0.43647 X –0.00597 X^2^

t-stat (53.1894) (24.3093) (-21.4892)

P-value (0.0000) (0.0000) (0.0000)

**Supplementary Table 7.** Information on model fittings, estimated CVPP and corresponding calculated F of the predicted equations of mean BMI at total, urban and rural area of Bangladesh in 2004, 2007, 2011 and 2014

| **Models** | **n** | **k** | **$\rho_{cv}^{2}$** | **Shrinkage** | **Cal. F(Tab. F=5.34 at 1% level with (2,32) d.f.)** |
| --- | --- | --- | --- | --- | --- |
| Equation 1 | 35 | 2 | 0.945334 | 0.00767648 | 304.22 |
| Equation 2 | 35 | 2 | 0.931990 | 0.00955026 | 241.59 |
| Equation 3 | 35 | 2 | 0.981782 | 0.00255828 | 942.85 |
| Equation 4 | 35 | 2 | 0.976128 | 0.00335223 | 715.99 |
| Equation 5 | 35 | 2 | 0.979246 | 0.00291441 | 825.81 |
| Equation 6 | 35 | 2 | 0.915156 | 0.01191414 | 190.68 |
| Equation 7 | 35 | 2 | 0.975814 | 0.00339634 | 706.51 |
| Equation 8 | 35 | 2 | 0.976139 | 0.00335060 | 716.35 |
| Equation 9 | 35 | 2 | 0.946648 | 0.00749188 | 312.08 |
| Equation 10 | 35 | 2 | 0.968403 | 0.00443697 | 537.28 |
| Equation 11 | 35 | 2 | 0.981130 | 0.00264976 | 909.78 |
| Equation 12 | 35 | 2 | 0.961423 | 0.00541715 | 437.35 |

# Note: n is the number of cases, and k is the number of predictors in the model; is the cross-validity prediction power (CVPP)
